# Soil amendments altered arbuscular mycorrhizal fungal communities in cadmium-contaminated vegetable fields

**DOI:** 10.3389/fmicb.2024.1470137

**Published:** 2024-11-18

**Authors:** Liang Li, Yanning Shi, Wangbiao Xia, Xiyang Wang, Zaijun Xin, Yingchun Liao, Xiaoyan Sun

**Affiliations:** ^1^Institute of Microbiology, Jiangxi Academy of Sciences, Nanchang, China; ^2^Nanchang Institute of Technology, Nanchang, China

**Keywords:** arbuscular mycorrhizal fungi, soil amendments, heavy metals, cadmium, vegetable fields

## Abstract

Soil amendments, including various types of fertilizers, are often used to control the uptake of heavy metals such as cadmium in cropping fields. The influence of these amendments on other members of the agroecosystem, such as arbuscular mycorrhizal fungi (AMF), remains less well investigated. Here, we established an experiment with the application of woody peat organic fertilizer and phosphate rock powder to examine its effects on AMF communities in two cadmium-contaminated vegetable crop fields (cucumber and pepper). We found that the application of phosphate rock powder enhanced soil phosphorus content, while the application of woody peat organic fertilizer enhanced soil nitrogen content, but neither influenced AMF abundance. We also found little influence of either amendment on measures of AMF diversity, except in one case where the Shannon index of diversity was lower in pepper fields amended with phosphate rock powder. We did, however, find significant shifts in the community composition and relative abundances of AMF taxa in the two vegetable fields, primarily as a result of shifts in the soil pH and nitrogen content.

## 1 Introduction

As a result of ongoing anthropogenic activities, agricultural soils are being continually degraded (Durdu et al., 2023). Notably, heavy metals are accumulating in agricultural soils globally because of increased uses of pesticides and fertilizers, as well as other industrial actions (Rashid et al., 2023). This heavy metal contamination can negatively influence crop production through a number of pathways, and can also influence human health. For example, cadmium (Cd) is a non-essential element for plant growth, but owing to its high chemical activity in soil, can be easily absorbed by plants and accumulate within plant tissues, ultimately posing risks to human health through consumption (McLaughlin et al., 2021). Levels of Cd have increased in agricultural soils in recent decades (Six and Smolders, 2014; Shi et al., 2019), and now reducing the Cd content and activity in soils, as well as decreasing its absorption and accumulation by plants, has become a priority for safe production of edible agricultural products (Mubeen et al., 2023).

For lightly and moderately polluted soils, a number of soil amendments, including organic material and phosphate, have been commonly applied to reduce the bioavailability of heavy metals in the soil through the mechanisms of adsorption, complexation and precipitation (Gao J. et al., 2023; Wang F. et al., 2022; Cui et al., 2023). While there has been work to understand the impact of these activities on farmland soil, such as the positive effects of reducing plant heavy metal content and crop yield (Feng and Cheng, 2023), less attention has been paid to the influence of these amendments on environmental and ecological risks, such as the impact of these measures on soil microbial community structure and their functions (Holland et al., 2018). For example, how these amendments might influence crop interactions with soil microbes, such as arbuscular mycorrhizal fungi (AMF) that form symbiotic relationships with over 70% of terrestrial plants (Brundrett and Tedersoo, 2018), obtaining carbon from plants in exchange for helping plants to absorb mineral nutrients and water (Smith and Smith, 2011).

In agriculture, AMF can promote crop yields while reducing the application of chemical fertilizers, controlling disease occurrence, and enhancing the host plant’s adaptability to drought, salinity and heavy metal pollution (Hao et al., 2022). AMF can also reduce the uptake and transport of Cd in crops through various mechanisms, including regulating plant and root growth, binding heavy metals with root and hyphal exudates, increasing antioxidant enzyme activities, interacting with soil beneficial microorganisms, and immobilizing heavy metals via extraradical hyphae as well as their improvement on soil aggregation (Wang, 2017; Wang H. et al., 2022; Moreno Jiménez et al., 2024). Studies on the application of AMF inoculant, combined with other remediation strategies, to mitigate Cd contamination in soils has shown promising results for various crops (Cakmak et al., 2023; Zulfiqar et al., 2023; Liu et al., 2024).

Given that AMF are widespread soil fungi in agricultural ecosystems, native AMF communities may also significantly influence the Cd remediation efficacy of soil amendments. Therefore, evaluating the interactions between soil amendments and native AMF communities is essential when developing strategies to mitigate Cd contamination. Findings from studies examining the effects of soil environmental changes on AMF under conditions that do not involve or explicitly consider Cd contamination remain inconclusive and highly context-dependent (van der Heyde et al., 2017; Mohamed et al., 2023). For example, some studies report that AMF abundance are enhanced by organic amendments or reduced by excessive phosphate, while others find more nuanced or contrasting outcomes (Treseder, 2004; Park et al., 2024). The diversity of outcomes highlights the complexity of AMF interactions with soil environments and the necessity for more targeted research that considers the specific conditions under which AMF are studied. This includes exploring how soil amendments can influence the structure and function of AMF communities in the context of Cd contamination.

Here, we used an experimental approach to explicitly investigate the effects of soil amendments on the AMF community in Cd contaminated vegetable fields. We examined changes in both soil chemistry and the AMF community, and their associations, to understand the primary factors influencing AMF community. We used two types of vegetable crops, pepper (*Capsicum annuum*) and cucumber (*Cucumis sativus*), and two types of soil amendments (phosphate rock powder and woody peat organic fertilizer). In all, our research will provide insights into the responses, protection and potential application of AMF communities in farmland soils.

## 2 Materials and methods

### 2.1 Site

Our study took place in Jiangxi Province, China (30°31′N, 115°56′E), characterized by a subtropical continental monsoon climate. The annual average temperature is 17.5°C and the average annual precipitation is 1,600 mm. The soil type in the area is typical red soil, with Cd levels ranging from 0.61 to 1.23 mg kg^−1^. According to the national risk control standard for soil contamination of agricultural land, the risk screening values for Cd contamination in vegetable fields are 0.3 mg kg^−1^ for soils with a pH below 7.5 and 0.6 mg kg^−1^ for soils with a pH above 7.5 (Chinese National Standardization Administration, 2018). Therefore this site is considered to be light to moderately Cd contaminated. The site of our field experiment is cultivated with various vegetables, including peppers, cucumbers, Chinese cabbage, cauliflower and tomatoes.

### 2.2 Experimental design

In June 2022, we established 13 plots of 1 m × 20 m in each of two adjacent land parcels (26 plots in total), one where peppers were cultivated and the other where cucumbers were cultivated. At each site, we established a completely randomized design with three treatments: (1) the addition of phosphate rock powder (P, with five replicates), with the particle size of 75 μm, consisting of 13.9% P_2_O_5_ and 0.033 mg kg^−1^ of Cd; (2) the addition of woody peat organic fertilizer (O, with five replicates), containing ≥45% organic matter, ≥10% humic acid, and ≥ 5% N + P_2_O_5_ + K_2_O, with a Cd content of 0.068 mg kg^−1^; (3) a control (C, no amendment was added, with three replicates). Both soil amendments were applied at a rate of 0.3 kg·m^−2^, following the application rates commonly used in local Cd-contaminated soil remediation practices.

After crops were harvested in October, we selected five random locations within each plot to collect soil cores. These locations were systematically distributed along the longer edges of each plot to ensure representative sampling. We determined a sampling depth of 20 cm based on the average tillage depth commonly employed in this vegetable fields. To collect an adequate volume of root segments for assessing mycorrhizal colonization, we used a large-diameter soil corer (10 cm). We thoroughly mixed the collected soil to form a single sample, with a total volume of ~3 kg. We picked at least 30 small root segments from each soil sample and preserved in a 50% v/v alcohol solution for subsequent analysis of mycorrhizal colonization. Then, we removed two 10 g soil samples which were frozen at −20°C. We used one of these samples for DNA sequencing and the other for the analysis of hyphal density. Finally, we used the remaining soil for chemical analysis after drying at room temperature.

By analyzing the Cd content in cucumber and pepper fruits (unpublished data), we observed that Cd concentrations in fruits from amended fields were consistently below the national food safety threshold of 0.05 mg kg^−1^ fresh weight. Additionally, while the application of soil amendments resulted in a Cd reduction of 7 to 18% compared to the control fields, this decrease, though notable, did not reach statistical significance (*p* > 0.05). These results highlight the potential, albeit limited, efficacy of soil amendments in mitigating Cd accumulation in edible plant tissues.

### 2.3 Soil chemistry

We analyzed soil organic matter using the potassium dichromate titration method; total nitrogen and available (alkali-hydrolyzable) nitrogen using a Kjeldahl nitrogen analyzer; total phosphorus and Cd by inductively coupled plasma optical emission spectrometry; available phosphorus extracted with hydrochloric acid-ammonium fluoride and determined by the molybdenum antimony colorimetric method; and pH by the potentiometric method with a water-to-soil quality ratio of 2.5:1.

### 2.4 AMF abundance

We estimated AMF abundance including both mycorrhizal colonization rate and hyphal length density using the methods in Brundrett et al. (1994). We counted AMF intraradical structures using the intersecting lines method (McGonigle et al., 1990) with 200 fields of view examined per sample and calculated mycorrhizal colonization rate (%) as the ratio of the number of fields of view containing AMF structures to the total number of fields. We counted the extraradical hyphal length using the grid intersection method (Tennant, 1975) with 100 fields of view observed per filter membrane. We calculated the total hyphal length on each filter using the formula:


$$h=\left[\left(\sum c\right)\times \left(11/14\right)\times a\right]\times \left[\pi \times r^{2}\right]/\left[\left(\sum s/100\right)\times g^{2}\right]$$

where *h* represent the hyphal length (mm), Σ*c* is the total number of intersections between hyphae and grid lines, Σ*s* was the total number of fields observed (100 in this study), *a* was the side length of each small square in a 10-part grid (0.05 mm in this study), and *g* was the side length of the grid (0.5 mm in this study). Finally, the hyphal length density was converted to the density per unit dry weight of soil based on the soil moisture content.

### 2.5 DNA extraction and sequencing

We used high-throughput sequencing to analyze the diversity and compositional shifts of AMF communities. To do so, we extracted total DNA from 300 mg of soil samples, from which we assessed the concentration and purity. We used a nested PCR to amplify the 18S rDNA small subunit (SSU) regions using the primer pairs AML1/AML2 (Lee et al., 2008) for the first amplification and AMV4.5NF/AMDGR for the second amplification (Sato et al., 2005). We recovered the PCR products, purified and eluted them, and used electrophoresis for detection, which we then quantified. DNA extraction and sequencing were performed by Shanghai Majorbio Bio-Pharm Technology Co., Ltd. (Shanghai, China), utilizing the Illumina MiSeq platform (Illumina, United States) for sequencing, with a minimum sequencing depth of 45,000 reads per sample.

### 2.6 Sequencing data processing

We quality-controlled the raw sequencing reads using Trimmomatic (Bolger et al., 2014) and assembled using FLASH software (Magoč and Salzberg, 2011). We removed chimeras using UCHIME software (Edgar et al., 2011). We clustered sequences subjected to quality control into Operational Taxonomic Units (OTUs) at a 95% similarity threshold using UPARSE (Edgar, 2013), and classified and annotated species using the RDP classifier,^1^ followed by comparison and identification with the MaarjAM database (Öpik et al., 2010).

### 2.7 Statistical analysis

We used one-way ANOVA to compare differences in soil chemical composition, AMF colonization rate and hyphal length density, and the OTU numbers and Shannon diversity index of AMF communities among different treatments for each of the two vegetable fields. To examine the relationship between AMF abundance and soil factors, we used Pearson correlation analysis. To compare the AMF community composition among treatments, we ordinated composition using non-metric multidimensional scaling (NMDS) based on Bray–Curtis distances using the *vegan* R package (Oksanen et al., 2017). We then used the ‘adonis’ function to conduct a permutational multivariate analysis of variance (PERMANOVA) to compare AMF community structure among the treatments. We employed a Variance Inflation Factor (VIF) analysis to test for collinearity among variables when analyzing the factors influencing community composition using the ‘vif’ function in the *car* package (Fox and Weisberg, 2019). Next, we used the ‘envfit’ function to fit the selected soil factors to the NMDS ordination. We used the Kruskal-Wallis H test to analyze the differences in relative abundance of the top 20 most abundant OTUs across all treatments in each vegetable field in response to the application of soil amendments compared to the control. Differences among groups were assessed using Scheffe *post hoc* tests. AMF families typically exhibit conserved functional traits and responses to environmental changes (Chagnon et al., 2013), which makes family-level analysis relevant for understanding broad ecological patterns. By integrating both family and OTU-level analyses, we aimed to achieve a more nuanced and comprehensive perspective on how soil amendments impact the AMF community. We used the *pheatmap* package (Kolde, 2019) to calculate the Pearson correlation coefficients between soil chemistry and the AMF families, as well as differences among the top 20 most abundant OTUs. We conducted all statistical analyses in R 4.1.0 (R Core Team, 2022).

## 3 Results

### 3.1 Soil chemistry

We found that the addition of woody peat organic fertilizer and phosphate rock powder influenced several soil chemical variables compared to control treatments in both vegetable fields (Table 1). Specifically, both total and available nitrogen content increased in both vegetable fields with the application of organic fertilizer, while available phosphorus content increased in the pepper field with the application of phosphate rock powder. Soil pH was inherently lower in cucumber fields than that in pepper fields. The addition of organic fertilizer led to lower pH in pepper, but not cucumber fields. Finally, we found no differences in other soil chemical variables among the three treatments or between the two types of vegetable fields.

**Table 1:** Effects of applying amendments on soil chemical characteristics.

Soil variables	Pepper			Cucumber		
	C	P	O	C	P	O
pH	6.9 ± 0.1^a^	6.8 ± 0.2^a^	5.6 ± 0.0^b^	6.2 ± 0.1^a^	6.1 ± 0.2^a^	6.0 ± 0.3^a^
TOC (g kg^−1^)	30.3 ± 2.5^ab^	24.3 ± 2.9^b^	38.3 ± 4.8^a^	24.6 ± 2.0^a^	21.8 ± 2.1^a^	28.3 ± 7.7^a^
Cd (mg kg^−1^)	0.8 ± 0.1^a^	0.7 ± 0.1^a^	0.5 ± 0.1^a^	0.8 ± 0.1^a^	0.9 ± 0.1^a^	0.5 ± 0.1^b^
TN (g kg^−1^)	1.7 ± 0.1^b^	1.6 ± 0.1^b^	2.4 ± 0.0^a^	1.3 ± 0.0^b^	1.3 ± 0.1^b^	2.2 ± 0.2^a^
AN (mg kg^−1^)	123.3 ± 15.1^b^	105.8 ± 11.1^b^	215.1 ± 9.8^a^	67.1 ± 11.3^b^	61.5 ± 5.8^b^	158.5 ± 35.2^a^
TP (g kg^−1^)	1.2 ± 0.2^ab^	1.5 ± 0.2^a^	1.0 ± 0.1^b^	1.2 ± 0.1^a^	1.3 ± 0.1^a^	1.2 ± 0.3^a^
AP (mg kg^−1^)	43.7 ± 4.0^b^	68.2 ± 3.5^a^	42.4 ± 3.2^b^	53.5 ± 4.5^a^	63.1 ± 3.5^a^	36.0 ± 3.3^b^
N:P	1.5 ± 0.2^b^	1.1 ± 0.1^b^	2.6 ± 0.2^a^	1.1 ± 0.1^b^	1.0 ± 0.1^b^	2.0 ± 0.3^a^

Values are means ± SE, with different letters indicating the significant differences (*p* < 0.05). C, control treatment; P, phosphate rock powder; O, organic fertilizer. TN, total nitrogen; AN, available nitrogen; TP, total phosphorus; AP, available phosphorus; N:P, total nitrogen-to-phosphorus ratio; Cd, total cadmium; TOC, total organic carbon.

### 3.2 AMF abundance

**Figure 1:**
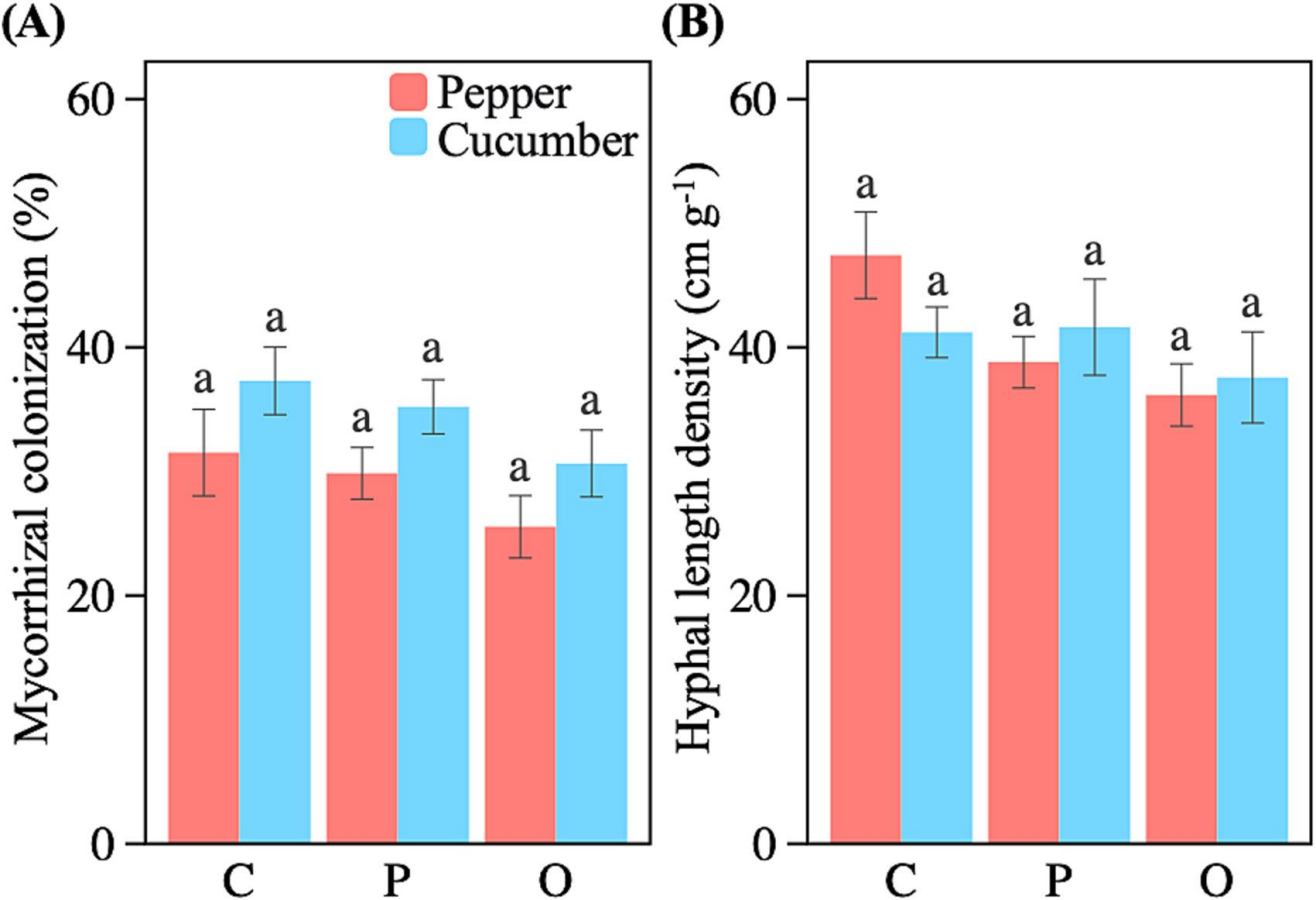
Effects of applying nutrient amendments on AMF colonization rate (A) and hyphal length density (B). Bars represent means ± SE, with different letters indicating significant differences (*p* < 0.05). C, control; P, phosphate rock powder; O, organic fertilizer.

There was no difference in mycorrhizal colonization rates in both pepper and cucumber following the addition of soil amendments compared to the control treatment (Figure 1A) (*F*_2,10_ = 0.9, *p* = 0.44; F_2,10_ = 1.5, *p* = 0.28). Likewise, we found no difference in the hyphal length densities between the treatments (Figure 1B) (*F*_2,10_ = 2.9, *p* = 0.10; *F*_2,10_ = 0.4, *p* = 0.70).

We found a significant negative correlation between mycorrhizal colonization rate and soil total nitrogen content (*r* = −0.48, *p* = 0.014), available nitrogen (*r* = −0.44, *p* = 0.024), as well as the soil nitrogen-to-phosphorus ratio (*r* = −0.50, *p* = 0.010). No significant correlation was found between the AMF abundance and the other soil variables (Table 2).

**Table 2:** Correlation between mycorrhizal colonization rate and hyphal length density and soil chemical variables across the three treatments and two vegetable fields.

Soil variables	*N*	Mycorrhizal colonization rate		Hyphal length density	
		Coefficient	*p-*value	Coefficient	*p-*value
pH	26	−0.03	0.869	0.21	0.300
TOC	26	−0.31	0.119	−0.05	0.808
Cd	26	0.26	0.202	0.13	0.537
TN	26	−0.48	**0.014**	−0.20	0.317
AN	26	−0.44	**0.024**	−0.13	0.524
TP	26	0.20	0.321	0.11	0.589
AP	26	0.15	0.477	−0.11	0.580
N:P	26	−0.50	**0.010**	−0.23	0.253

TN, total nitrogen; AN, available nitrogen; TP, total phosphorus; AP, available phosphorus; N:P, total nitrogen-to-phosphorus ratio; Cd, total cadmium; TOC, total organic carbon. The bold *p*-values indicate the significant differences (*p* < 0.05).

### 3.3 AMF community

We found a total of 1,567,232 optimized amplicon sequences from the 26 plots. After subsampling to a minimum sample sequence of 45,829, we assigned a total of 1,191,554 sequences to 193 taxa belonging to 7 families and 10 genera within the Glomeromycota phylum. Of these, 125 OTUs were identified in pepper fields and 149 OTUs were identified in cucumber fields. Across both vegetable crops, members of the Glomeraceae were the most dominant (76.3% of all sequences), followed by Paraglomeraceae (14.4%), Archaeosporaceae (7.4%) and Gigasporaceae (1.5%). These four families together comprised 99.5% of all sequences, while members of the Claroideoglomeraceae, Acaulosporaceae, and Ambisporaceae comprised less than 0.5%.

**Figure 2:**
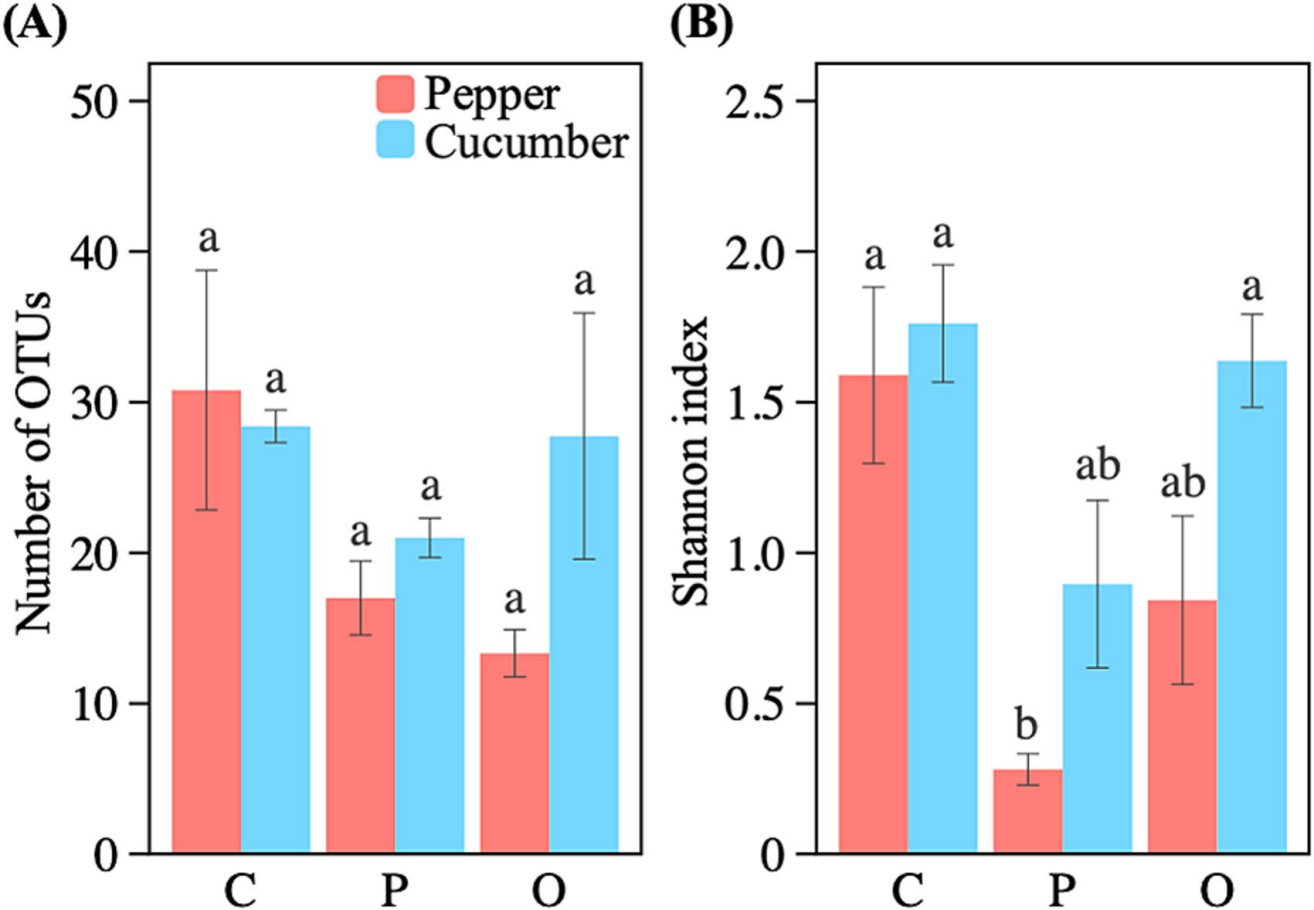
Effects of applying nutrient amendments on AMF OTU richness (A) and Shannon diversity index (B). Bars represent means ± SE, with different letters indicating significant differences (*p* < 0.05). C, control; P, phosphate rock powder; O, organic fertilizer.

The effects of soil amendments on the *α*-diversity of AMF communities varied with the type of amendments and vegetable species (Figure 2). We found that neither soil amendment influenced the number of AMF taxa in the soils of both vegetable fields (Figure 2A). For the Shannon index, we found a significant decrease in the pepper field after the addition of phosphate rock powder, but no such effect in the cucumber field, or with the application of organic fertilizer in either field (Figure 2B).

**Figure 3:**
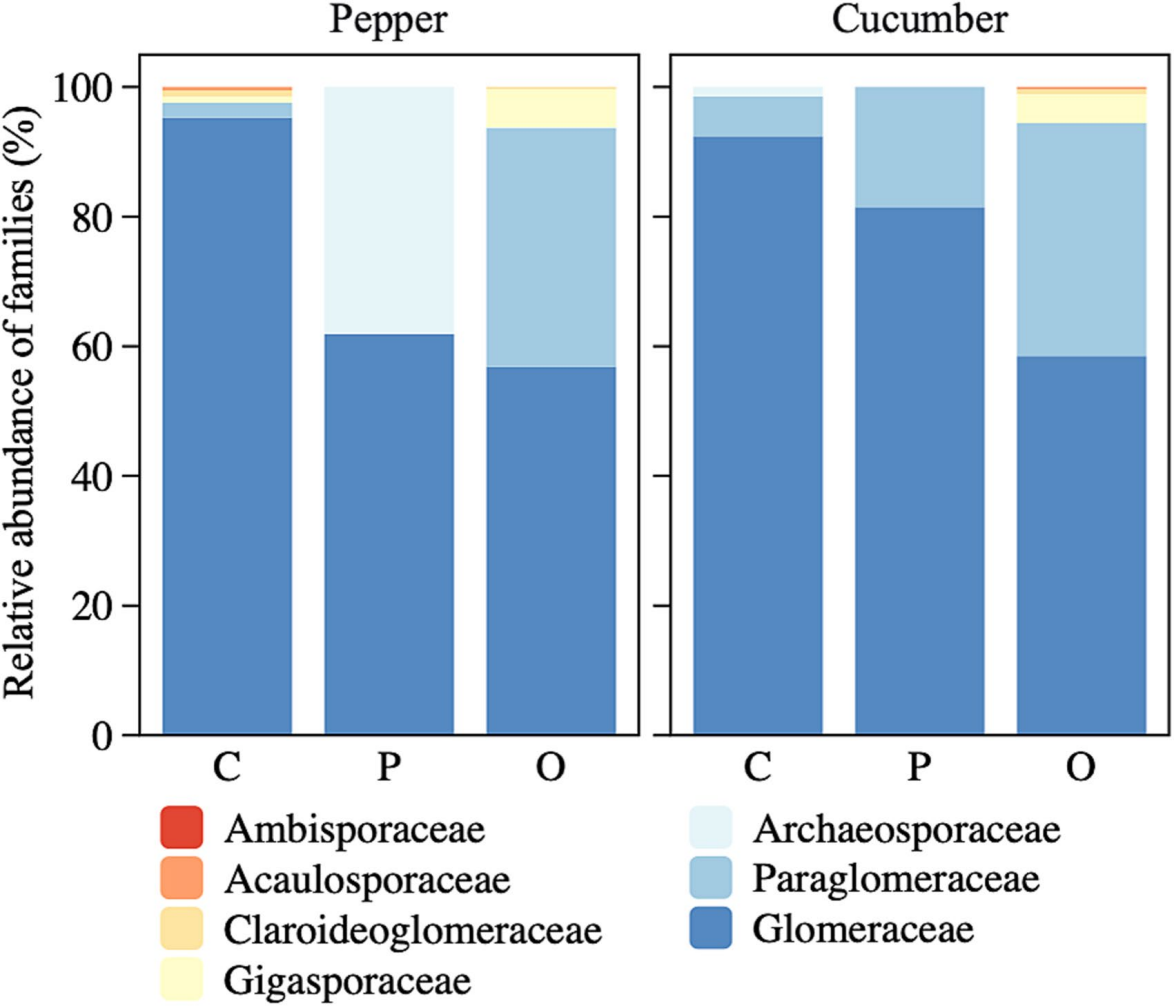
Effects of applying amendments on the relative abundance of AMF families. C, control treatment; P, phosphate rock powder; O, organic fertilizer.

At the family level, we found that the application of soil amendments led to a decrease in the relative abundance of the most dominant Glomeraceae in both vegetable fields (Figure 3). In the pepper field, Archaeosporaceae, which were absent in the control treatment increased to more than 35% in response to the addition of both amendments. Likewise, the relative abundance of Gigasporaceae, which was rare in the control treatment increased with the addition of organize fertilizer in both vegetable fields. In the cucumber field, the relative abundance of Paraglomeraceae increased substantially from the control treatment to the addition of both amendments.

**Figure 4:**
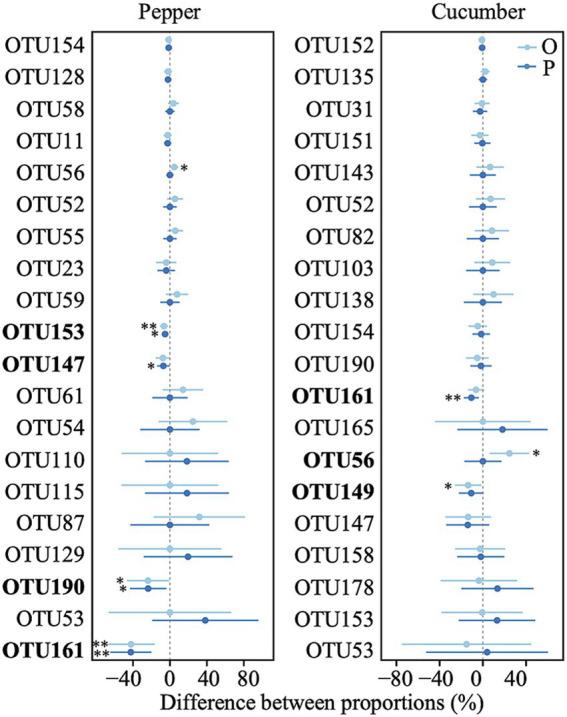
Differences in relative abundances of the dominant OTUs in each of the two vegetable fields applying soil amendments compared to the control. P, phosphate rock powder; O, organic fertilizer. *p < 0.05, **p < 0.01.

Among the top 20 most abundant OTUs in the pepper field, four decreased in relative abundance after the addition of phosphate rock powder, while two decreased and one increased after the addition of organic fertilizer (Figure 4). In the cucumber field, one taxon decreased in response to phosphate rock powder addition, and one taxon increased and one decreased in response to organic fertilizer (Figure 4).

**Figure 5:**
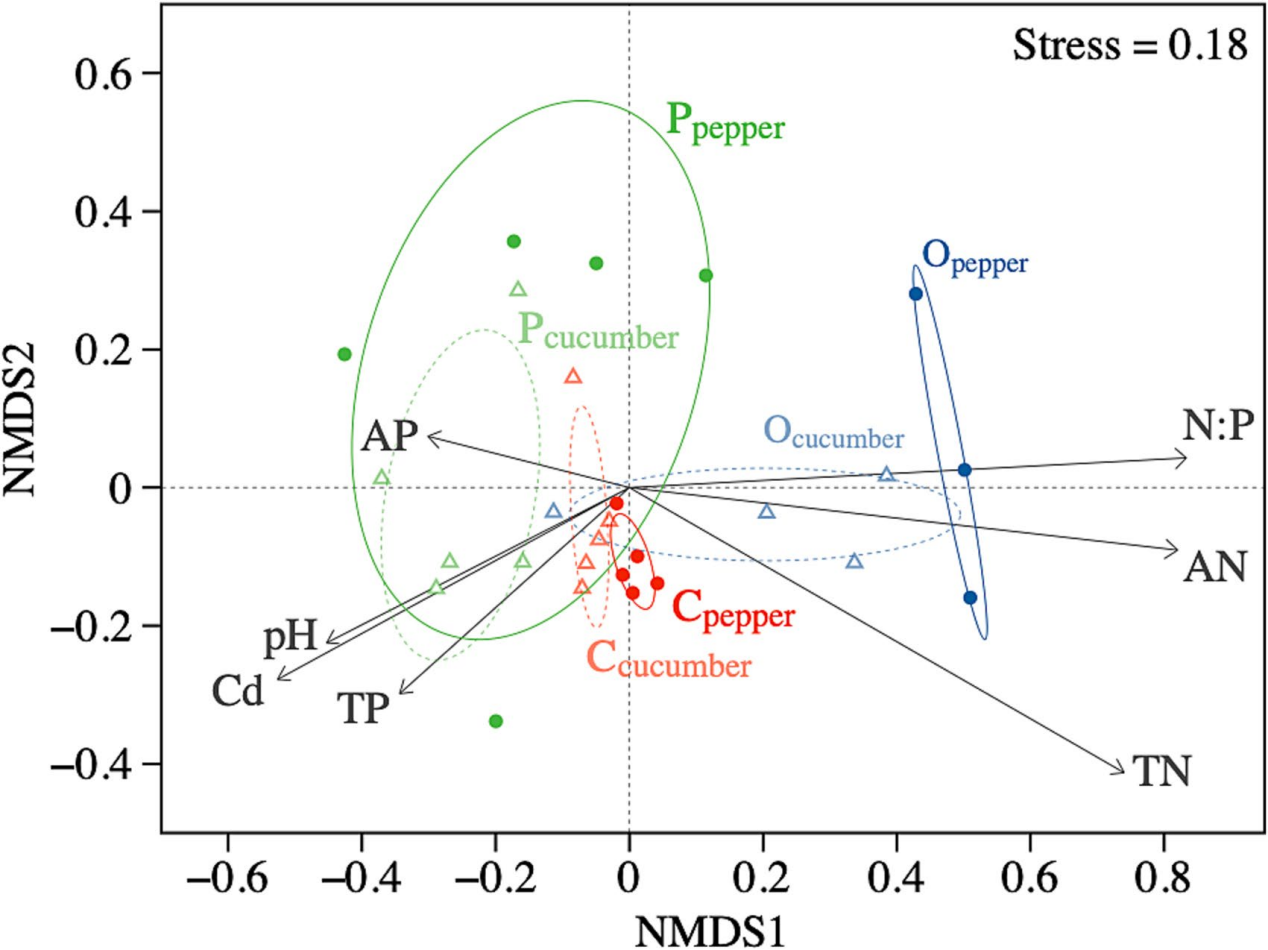
NMDS ordination showing changes in AMF community composition. Points represent each replicate of each treatment, while ellipses describe 95% confidence intervals for a given treatment. Arrows indicate the direction and degree of correlations between soil chemical characteristics and the NMD axes. C, control treatment; P, phosphate rock powder; O, organic fertilizer. TN, total nitrogen; AN, available nitrogen; TP, total phosphorus; AP, available phosphorus; N: P, total nitrogen-to-phosphorus ratio; Cd, total cadmium.

We found significant differences in the composition of the AMF community among the different vegetable fields and soil amendment treatments (*p* < 0.01). These differences were predominantly associated with variation in soil nitrogen and the nitrogen-phosphorus ratio (Figure 5) (*p* < 0.001). Other soil factors significantly associated with changes in AMF community structure included soil Cd content (*p* < 0.01) and pH (*p* < 0.05). In contrast, variations in soil phosphorus had a relatively minor contribution to these differences.

**Figure 6:**
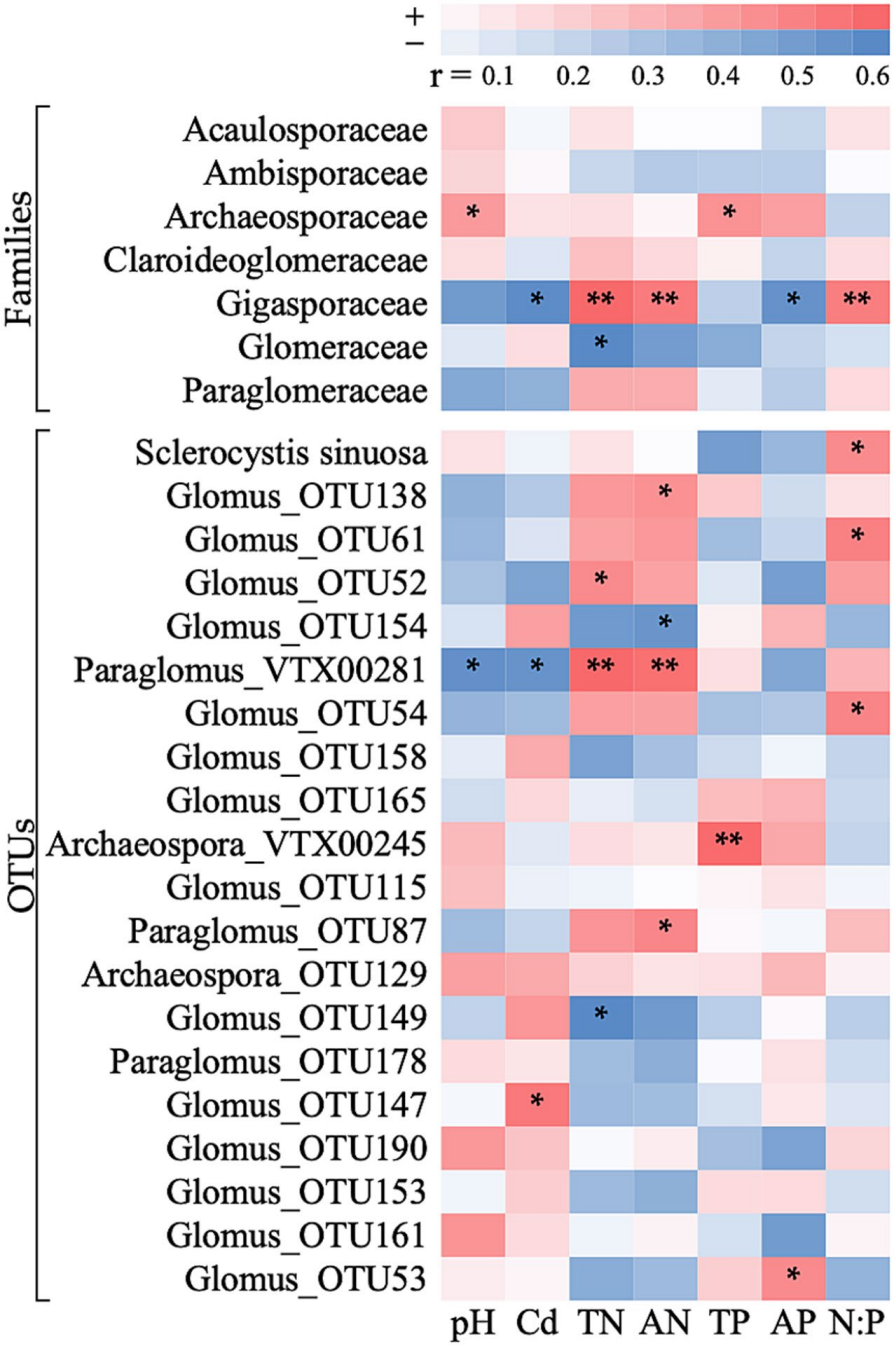
Correlation heat map between AMF families and dominant OTUs and soil chemistry. The colors indicate the correlation coefficient (*r*). TN, total nitrogen; AN, available nitrogen; TP, total phosphorus; AP, available phosphorus; N:P, total nitrogen-to-phosphorus ratio; Cd, total cadmium. *p < 0.05, **p < 0.01.

At the family level, the difference in relative abundance of the most abundant taxa, Glomeraceae, was significantly correlated with soil total nitrogen. The relative abundance of Gigasporaceae was significantly affected by several factors including Cd, total nitrogen, available nitrogen, available phosphorus content, and the nitrogen-phosphorus ratio. The Archaeosporaceae was influenced by soil pH and total phosphorus content (Figure 6). At the OTU level, of the top 20 most abundant OTUs, 12 showed significant correlations with soil factors (Figure 6). While most (11/12) of these OTUs was associated with only one soil factor, one was associated with four soil factors. Of the soil factors, available nitrogen was most strongly associated with variation in the most OTUs (Figure 6).

## 4 Discussion

Overall, it was not surprising that the application of organic fertilizer and phosphate rock powder as soil amendments led to significant increases in soil nitrogen and phosphorus levels (Table 1). The increase in soil nutrients could allow plants to rely more on their own root system for nutrient acquisition, reducing their dependence on AMF (Treseder, 2004; Singh et al., 2022) because the carbon costs of AMF associations may outweigh the nutritional benefits they provide (Johnson, 2010). However, despite the significant negative correlation between soil nitrogen and AMF abundance, we found no influence of the application of soil amendments on mycorrhizal colonization rates and hyphal length density (Figure 1). This is likely because the ambient levels of nitrogen and phosphorus are high in the soil, suggesting that nutrient acquisition may not be a primary role of AMF in symbiosis with plants. Indeed, the positive impacts of AMF on soil aggregation, water uptake and transport, and disease resistance can also play an important role in the plant-AMF symbiosis, but be less influenced by nutrient availability (Delavaux et al., 2017). Moreover, the relatively high ambient total phosphorus and organic carbon in the soil may have mitigated their responses to the common amounts of amendment addition (7% of total phosphorus and 2% of total organic carbon). This could partly explain the lack of significant changes in AMF abundance.

Despite the lack of response of AMF abundance to soil amendments, we did find lower Shannon diversity (but not richness) of AMF communities after applying phosphate rock powder. The adverse effects of nutrient additions on AMF diversity are consistent with results in many other ecosystems, including grasslands (Santos et al., 2006), alpine meadows (Liu et al., 2012), tropical rainforests (Camenzind et al., 2014), and other agroecosystems (Lin et al., 2012; Gosling et al., 2013). This is also consistent with the NMDS ordination results which indicated that applying phosphate rock powder led to a phylogenetically more dispersed AMF community (Figure 5). This community is more likely to be structured by competition, permitting taxa with various traits to respond to nutrient variations in different ways (Webb et al., 2002). However, the lack of response of AMF diversity to the nitrogen inputs from organic fertilizer may have been because the differential responses of AMF taxa to nitrogen and phosphorus addition (Camenzind et al., 2014).

Even when there was no influence of amendments on abundance or richness of AMF, we did consistently find shifts in the AMF community structure among treatments (Figure 5). Correspondingly, at both the family and OTU levels, there were notable changes in the relative abundance of dominant groups after the application of soil amendments, leading to increases of some taxa and decreases of others (Figures 3, 4). Different AMF taxa vary in their allocation to the intraradical structures that obtain carbon from plant roots and extra-radical hyphae that are responsible for obtaining nutrients from the soil (Maherali and Klironomos, 2012). As a result, taxa with a higher proportion of intraradical allocation (such as the Glomeraceae) tend to be more competitive in carbon resource acquisition (Chagnon et al., 2013), and thus tend to be more dominant. However, while we found that Glomeraceae dominated in the control treatment, their dominance diminished with soil amendments, while the abundance of Gigasporaceae, which has a greater advantage in acquiring soil nutrient resources, increased (Figure 3). This suggests that processes other than the availability of soil nutrients more likely regulates AMF community structure. Furthermore, it is possible that the effects of soil amendments on certain AMF groups may become more pronounced over an extended period. Due to the constraints imposed by the vegetable cropping system, the duration of our study was limited to 4 months, which represents one of the key limitations of this research.

Although we cannot full disentangle mechanisms leading the observed shifts in AMF community composition with the application of organic fertilizer, the declines in soil pH, observed specifically in pepper fields, may play an important role. Soil pH regulates both nutrient availability (Hartemink and Barrow, 2023) and AMF growth and reproduction (Wang et al., 1993; Van Aarle et al., 2002). As a result, AMF taxa with variation in nutrient and growth traits (Hart and Reader, 2002; Koch et al., 2017) can shift in their relative abundances under different levels of soil pH (Davison et al., 2021; Bodenhausen et al., 2023), such that soil pH may be a key driver of changes in AMF community composition (Figures 5, 6). A lack of pH change under organic fertilizer addition in cucumber fields suggests that crop-specific factors such as root exudates, nutrient uptake patterns, or differential microbial activity might mediate the soil’s response to amendments (Vives-Peris et al., 2020; Baggs et al., 2023; Shu et al., 2023). This discrepancy highlights the complexity of plant–soil-microbe interactions and underscores the importance of considering crop-specific responses when evaluating the effects of soil amendments.

At our study, not only was the ambient nitrogen and phosphorus content in the soil high, but so was the content of Cd (average of 0.7 mg/kg), leading to high risk for Cd pollution (Chinese National Standardization Administration, 2018). Although the response of AMF to variation in Cd content is mixed (Weissenhorn et al., 1993; Krishnamoorthy et al., 2015), we found no evidence for an influence of Cd on AMF abundance. However, we did find an influence of Cd on the composition and structure of AMF communities (Figures 5, 6). While we cannot explicitly disentangle the mechanisms underlying this effect, it is likely related to the differential responses of different AMF taxa to Cd (Gai et al., 2018; Chen et al., 2023). For example, the growth of Gigasporaceae family was markedly inhibited by Cd exposure (Figure 6). Existing evidence suggests that AMF communities in low P or high nitrogen-to-phosphorus ratio environments tend to favor Gigasporaceae species likely due to their competitive life history traits (Johnson et al., 2003; Chagnon et al., 2013). This is consistent with the significant increase in their abundance following the addition of organic matter in this study, whereas their response to phosphate addition was negligible (Figure 3). These findings suggest that the response of Gigasporaceae to varying resource inputs may be largely unaffected by the inhibitory effects of Cd, indicating a potential resilience of this family to heavy metal stress in specific environmental contexts. While the two soil amendments did not significantly influence the total Cd concentration in the soil, they have the potential to mitigate plant Cd uptake by decreasing Cd bioavailability (Wang F. et al., 2022). However, the absence of data on variations in available Cd content constrains our ability to comprehensively assess the extent to which these amendments may have impacted AMF abundance and community composition through the modulation of Cd bioavailability.

The compositional shifts of AMF communities we observed indicate that the use of soil amendments can have certain ecological impacts and/or risks (van der Heijden et al., 1998; Rillig and Mummey, 2006; Hawkins et al., 2023). Specifically, the increased abundance of Gigasporaceae in response to organic matter addition may enhance nutrient use efficiency in crops, improve soil aggregation, and boost carbon sequestration due to their significant investment in extraradical hyphae (Hart and Reader, 2002; Chagnon et al., 2013). In contrast, a decline in Glomeraceae could increase the risk of excessive Cd accumulation in agricultural products, as they generally demonstrate higher remediation efficiency than Gigasporaceae (Ferrol et al., 2009). Given the significant gaps in our understanding of the functional diversity of AMF communities, it remains challenging to predict the specific consequences of these changes (Wang and Rengel, 2024; Martin and van der Heijden, 2024) and how these changes in AMF communities will influence production in heavy metal-contaminated farmland. Nevertheless, the application of AMF inoculants on Cd contaminated farmland soils is considered an effective strategy to reduce plant Cd uptake and enhance crop yields (Liu et al., 2018; Gao Y. et al., 2023). Therefore, the practical application of our study lies in our ability to select representative taxa with differential responses to soil amendments, then explore their roles in reducing plant Cd uptake, increasing plant yields and improving soil aggregation, and to ultimately identify fungal strains with potential for widespread application. However, we did find that AMF responses to soil amendments differed between the two different kinds of crops in our study, underscoring the need for considering crop-dependent strategies for AMF inoculation in agricultural soils, where targeted AMF inoculation could be tested for its efficacy in reducing Cd uptake and ensuring food safety.

## 5 Conclusion

We examined the response of AMF communities to the application of soil amendments intended to allow safer utilization of crops in Cd contaminated vegetable fields. We found that in two types of Cd-contaminated vegetable fields (peppers and cucumbers), the AMF community composition and structure changed with the addition of soil amendments, but no change in the overall abundance. Interestingly, this response was primarily associated with changes in soil nitrogen and the nitrogen-to-phosphorus ratio, rather than phosphorus itself, reflecting the important role of the absolute levels of nutrients in regulating AMF communities. Additionally, we preliminarily identified certain AMF taxa that were more responsive to soil amendments. These findings provide valuable insights into exploring and identifying more efficient AMF strains for Cd remediation. However, further research is needed to elucidate the mechanisms underlying these responses and determine whether the selected taxa play specific roles in regulating plant heavy metal uptake. Long-term field trials across a wider range of crops and soil conditions are crucial to ensure the scalability and reliability of these strategies for broader agricultural applications.

## Data Availability

The datasets presented in this study can be found in online repositories. The names of the repository/repositories and accession number(s) can be found: https://www.ncbi.nlm.nih.gov/, PRJNA1131945.

## Footnotes

^1^ https://github.com/rdpstaff/classifier
